# Editorial for the Special Issue on Neural Electrodes: Design and Applications

**DOI:** 10.3390/mi10070466

**Published:** 2019-07-12

**Authors:** Joseph J. Pancrazio, Stuart F. Cogan

**Affiliations:** Department of Bioengineering, The University of Texas at Dallas, 800 W. Campbell Road, BSB 13.633, Richardson, TX 75080, USA

Neural electrodes enable the recording and stimulation of bioelectrical activity from the nervous system. This technology provides neuroscientists and clinicians with the means to probe the functionality of neural circuitry in health and modulate activity in disease states. In their simplest forms, neural electrodes can be viewed as collections of wires or exposed electrical contacts in an insulating substrate. The exposed electrical contact can vary in size depending on the application. For conventional therapeutic devices, the neural electrode sites are relatively large, allowing the electrical stimulation of a volume of tissue to effect neuromodulation. For example, neural electrodes can deliver therapeutic stimulation for the relief of debilitating symptoms associated with Parkinson’s Disease, a disorder characterized by a deficit in dopaminergic neurons, via deep brain stimulation (DBS). The neural electrodes for DBS consist of a relatively small number of metallic contacts along a single shaft. 

In comparison, microelectrode and even ultramicroelectrode-based devices, which offer the opportunity for a more intimate interface with the nervous system tissue, are 1–3 orders of magnitude smaller in dimension, and the field is driving towards high spatial densities of electrode sites. Indeed, from a historical standpoint, basic and applied neuroscience has made significant strides from insights derived from small, yet fragile and unstable neural electrodes. At small dimensions, neural electrodes can stimulate relatively small tissue volumes to provide selectivity or allow the measurement of activity from a single “unit” or presumptive individual neuron for decoding purposes. While the performance consistency of the microelectrode–tissue interface has been problematic, the experimental results have inspired efforts to develop motor and sensory neuroprosthesis as well as provided fundamental insights into brain circuitry. Commercially available arrays of microelectrodes include bundled microwires, multi-shank silicon-based Utah (or Blackrock) arrays, and the Michigan (or Neuronexus) single and multi-shank devices. A significant obstacle to large-scale implementation is the “many wire problem”, i.e., how many wires can one logistically implement across the skull? Consider, for example, a mouse skull with a surface area of approximately 100 mm^2^. For the smallest Omnetics connector, often used in basic science and pre-clinical neuroscience studies, the pitch between electrical contacts is 0.635 mm. The largest number of connection points for the entire 100 mm^2^ surface area would be 225, far fewer than the 75 million neurons in a mouse brain. While multiplexing and wireless transmission will likely ameliorate or avoid altogether cabling and connector issues in future devices for clinical applications and long-term behavioral studies, robust cabling is non-trivial, and stresses created by cable bundles remain a major limitation for high density microelectrode devices, affecting positioning and stability.

Furthermore, there are failure mechanisms that include both biotic and abiotic processes. As reviewed by Campbell and Wu [1], the biotic mechanism is believed to originate from the chronic foreign body response following initial implantation, and downstream processes that have features consistent with CNS trauma and neurodegeneration. There is evidence implicating the mechanical mismatch between the nervous system tissue and the neural electrodes as a source of the tissue response [2]. The Young’s modulus for brain tissue is approximately 7 orders of magnitude softer than silicon, a common neural microelectrode structural material. Neural probes that either consist of materials that soften on implantation [2,3] or have extremely small cross-sectional areas resulting in device flexibility [4] show diminished tissue response. From an abiotic perspective, electrical connectors, never intended by design for biological experiments, are prone to fail due to repeated plugging and unplugging, a problem that plagues neural recordings in animal models. The combination of optogenetics with neural recording and stimulation paradigms creates new challenges for ensuring that heat dissipation during illumination is minimized to reduce the likelihood of tissue damage. Lastly, the age of bioelectronic medicines has brought the realization that the viscera are subject to neuromodulation and that new electrode technologies will be required to unravel functional neural connectivity. Multidisciplinary approaches are the key, and so this Special Issue on “Neural Microelectrodes: Design and Applications” showcases 22 research papers and review articles that leverage new perspectives from fields including tissue biomechanics, material science, and biological mechanisms of inflammation and neurodegeneration that are critical to advancing the technology. The papers in this Special Issue explore the following aspects of neural electrodes design and application in the following theme areas: (1) biomaterials, (2) enabling technology and fabrication, and (3) the biology of the interface.

1. Biomaterials. Hess-Dunning and Tyler [3] report that neural probes can be fabricated from a mechanically-adaptive polymer that is stiff prior to insertion, but rapidly transitions in vivo to an extremely compliant state (~10 MPa). These devices are fabricated from a nanocomposite derived from a soft poly(vinyl acetate) (PVAc) matrix polymer with a percolated network of high-aspect-ratio cellulose nanocrystals harvested from tunicate mantles. The nanocomposite supports embedded Parylene-C-coated Au leads to produce stable electrochemical properties in vivo and well-resolved single units from probes implanted chronically for 4 months in rat cortex. Stiller et al. [5] describe the chronic recording and electrochemical performance of fully encapsulated shape memory polymer (SMP) microelectrode arrays in rat cortex. These single shank devices, fabricated from conventional thin film polymer processing, are based on thiol-ene/acrylate SMP that softens by an order of magnitude once implanted. After 4 months in vivo, single units were prominent, and the neuroinflammatory profile determined by immunohistochemistry was modest, suggesting the feasibility of SMP-based devices. Shoffstall et al. [6] describe a comprehensive analysis of the neuroinflammatory response of SMP-coated silicon structures and compare the surface properties to uncoated silicon structures. After 4 months in rat cortex, the non-softening thiol-ene SMP devices appeared to produce a histological profile similar to the silicon controls, although astrocytic scarring was reduced for the SMP-coated probes. Rihani et al. [7] present early stage work showing that another smart polymer, specifically liquid crystal elastomer with shape-changing characteristics, may be a promising neural interface material. Based on in vitro studies, they report that the material is neither cytotoxic nor likely to affect neuronal electrophysiologic behavior. Furthermore, they demonstrate that functional multi-layer devices consisting of arrays of electrodes can be fabricated and are electrically stable under in vitro conditions. 

Rather than having a device consist of material that changes its modulus, Kil et al. [8] demonstrate that dextran can be used to temporarily stiffen flexible probes fabricated from Parylene-C to enable insertion into rat somatosensory cortex without buckling. They characterized the rapid dissolution rate as a function of molecular weight and probe surface area, showing that a 37 µm thick coating of 40 kDa dextran provides a dissolution time of approximately 4 min, well within the typical time period needed to insert a cortical probe. Histological analyses of dextran-coated probes implanted for 4 months demonstrated little or no neuroinflammatory response, indicating that dextran is a practical option for temporarily stiffening ultrasmall and highly flexible probes for insertion. Deku et al. [4] describe the fabrication and in vivo performance of ultrasmall microelectrode arrays that leverage amorphous silicon carbide (a-SiC), an insulating material known to be non-corrosive and fracture-resistant. Prototype a-SiC intracortical implants fabricated containing 8–16 single shanks with a thickness of 6 µm penetrated rat cortex without an insertion aid or transient coating, and single unit recordings were well-resolved from sputtered iridium oxide-coated electrode sites. Recognizing that delamination is one of the means by which implanted multi-layer devices fail, Bernardin et al. [9] report on the early stage development of a monolithic probe that makes use of a crystalline form of silicon carbide (SiC). Capitalizing on SiC as a chemically inert semiconductor, the device structure was micromachined from p-type SiC with conductors created from n-type SiC, simultaneously providing electrical isolation through the resulting p-n junction. Electrical characterization of the electrode sites showed high-performance p-n diode behavior, with typical turn-on voltages of ~2.3 V and reverse bias leakage below 1 nA_rms_ with impedances suitable for extracellular recording.

2. Enabling Technology and Fabrication. Sridharan et al. [10] show that rat peripheral nerve can be wirelessly stimulated using volume-conducted AC fields directed towards implanted Schottky microdiodes coupled to cuff electrodes. Muscle twitches monitored by electromyography could be elicited by 500 kHz AC fields for as brief as 1 ms in duration. While there may be limitations in terms of the number of selective channels, this approach is promising for a range of “bioelectronics medicines” applications. Straka and colleagues [11] report that electrochemical impedance spectroscopy (EIS) performed in vivo can provide insights into degradation kinetics and mechanisms associated with implanted Utah arrays in peripheral nerve. Capitalizing on electrode-electrolyte equivalent circuit representation for Pt and Pt/Ir. microelectrodes based on the Randles circuit model, distinct classes of EIS become apparent that implicate the emergence of a glial scar from the neuroinflammatory response, parasitic capacitance pathways, lead-wire breakage or loss of electrode tip metallization, or electrode insulation degradation. While DBS systems typically operate in an open loop, always on mode, Vajari et al. [12] describe the design of a hybrid DBS probe, comprised of glassy carbon electrodes on a polyimide substrate, which couples electrical stimulation and local field potential recording with fast scan cyclic voltammetry (FSCV). FSCV offers the possibility of real time measurements of dopamine for use as a feedback signal for a closed loop DBS system. In vitro characterization of the prototype device showed sub micromolar sensitivity to dopamine and minimal artifacts during magnetic resonance imaging. Future preclinical work will be necessary to characterize the stability of glassy carbon electrode measurements in vivo. 

Nicolai et al. [13] provide a characterization of motion artifacts associated with real world, in vivo use of ultra-small, flexible 7-µm diameter carbon fiber electrodes. They show that motion artifacts generated by the movement of electrodes during electrophysiological recordings and fast-scan cyclic voltammetry are difficult to distinguish from the characteristic action potential and neurochemical signals. They hypothesize that motion alters the electrode/electrolyte interface by affecting the electric double layer to trigger the artifacts. Sharma et al. [14] address the “many wire” problem with the design and testing of a novel electronic architecture for intracortical neural recording. By integrating mixed-signal feedback, windowed integration sampling, and a successive approximation analog-to-digital converter, they show promising results from a 180 nm CMOS integrated circuit prototype capable of multiplexing high channel count microelectrode arrays. Xu et al. [15] offer a technological solution to the problem of simultaneous recording and stimulation. They propose a novel bidirectional neuromodulation system-on-chip, which includes a frequency-shaping neural recorder and a fully integrated neural stimulator with charge balancing capability. A prototype device was fabricated and shown to be capable of achieving simultaneous electrical stimulation and recording on the same nerve preparation in vivo. 

To provide enhanced control during optogenetic studies, Goncalves et al. [16] describe a hybrid device combining optical stimulation and neural recording, which is capable of monitoring the heat generated during light exposure. A proof-of-concept device, with double-sided function: on one side, an optrode with LED-based stimulation and Pt recording sites on one side of the probe and with a Pt-based thin-film thermoresister for temperature measurement on the opposite side, was fabricated and characterized. The silicon-based device showed good recording and optical features with suitable optical power delivery and a high-resolution temperature monitoring, indicating that this optrode approach may be useful for limiting tissue damage from excessive heating. Scholvin et al. [17] report on a scalable, modular strategy for fabricating high-density 3D neural probes. They demonstrate a 3D probe constructed from individual 2D components where there are arrays of shanks consisting of densely packed, at a 40 µm pitch, electrode sites. The probes are assembled using mechanical self-locking and self-aligning techniques followed by electroless nickel plating to establish electrical contact. By combining scalable 3D design and high-density recording sites, this fabrication approach may enable new classes of devices capable of large-scale neural recording to elucidate the functional connectivity of the brain. Hoch et al. [18] address the problem associated with neural probe connector failure, which is triggered by excessive forces generated by repeated plugging/unplugging between recording sessions. They developed a new magnetic connector system that uses multiple magnet pairs and spring-suspended electrical contact pads realized using micro-electromechanical systems technologies to achieve reliable self-alignment of the connector parts at ±50 µm with negligible connection forces.

3. Biology of the Interface. Campbell and Wu [1] provide a comprehensive review of the tissue response to devices implanted within the brain and illustrate how both biotic and abiotic processes result in failure in bioelectrical performance. They discuss the tissue response to electrode implantation and the associated molecular pathways that act on acute and chronic timescales. In addition, they review current strategies to minimize the tissue response to enhance device reliability. Genetic tools offer an opportunity to target critical proteins that may have pivotal roles in neuroinflammatory response at the tissue–device interface. Winter et al. [19] characterize three different approaches for modifying gene expression at the tissue–device interface: viral-mediated overexpression, siRNA-enabled knockdown, and cre-dependent conditional expression. By making use of an implantable neural probe that incorporates microfluidics, they successfully delivered the vectors along the length of the device based on protein expression levels, indicating that this approach can be used to modulate various molecular pathways. In a meta-analysis derived from the published literature, Stiller et al. [2] explore the relationships between the neuroinflammatory responses to implanted devices that vary with respect to structural device stiffness, which is a function of both material modulus and cross-sectional area. By incorporating data from nine published studies, spanning a wide range of implant dimensions and materials, it was determined that the severity of the immune response, within the first 50 µm of the device, is highly correlated with device stiffness, as opposed to either the device modulus or cross-sectional area independently.

To complete the Special Issue, three papers provide insight into the past and future of neural electrodes. Shokoueinejad et al. [20] provide a historical perspective on the use and evolution of electrocorticography for a wide range of experimental and clinical applications. These grids, especially those that are 10–100 s of microns in dimension, offer the advantage of high-density neural signal acquisition and stimulation capabilities. In addition, this approach is considered relatively minimally invasive based on a muted neuroinflammatory response, at least compared to penetrating microelectrode arrays. Similar to electrocorticography arrays, devices suitable for recording the activity of the gut have been recently considered to shed light on the enteric nervous system. Barth et al. [21] describe the opportunity for advancing enteric neuroscience offered by single-unit recording capabilities in awake animals, using flexible conformal grids, and identify the primary design challenges for such devices. Finally, Kozai [22] provides a historical perspective on the progress and challenges for implantable neural electrodes, arguing that understanding the complexities associated with reliable chronic neural interfaces requires simultaneous proficiency in multiple scientific and engineering disciplines.

We extend our appreciation to all of the authors for their excellent contributions to this Special Issue of Micromachines. We also thank the peer referees who provided in depth, thoughtful, and rapid reviews of all of the manuscripts. Lastly, we acknowledge the dedicated journal staff of the editorial office who professionally and gracefully kept the entire process on track.

## References

[1] Campbell A., Wu C. (2018). Chronically Implanted Intracranial Electrodes: Tissue Reaction and Electrical Changes. Micromachines.

[2] Stiller A.M., Black B.J., Kung C., Ashok A., Cogan S.F., Varner V.D., Pancrazio J.J. (2018). A Meta-Analysis of Intracortical Device Stiffness and Its Correlation with Histological Outcomes. Micromachines.

[3] Hess-Dunning A., Tyler D.J. (2018). A Mechanically-Adaptive Polymer Nanocomposite-Based Intracortical Probe and Package for Chronic Neural Recording. Micromachines.

[4] Deku F., Frewin C.L., Stiller A., Cohen Y., Aqeel S., Joshi-Imre A., Black B., Gardner T.J., Pancrazio J.J., Cogan S.F. (2018). Amorphous Silicon Carbide Platform for Next Generation Penetrating Neural Interface Designs. Micromachines.

[5] Stiller A.M., Usoro J., Frewin C.L., Danda V.R., Ecker M., Joshi-Imre A., Musselman K.C., Voit W., Modi R., Pancrazio J.J. (2018). Chronic Intracortical Recording and Electrochemical Stability of Thiol-ene/Acrylate Shape Memory Polymer Electrode Arrays. Micromachines.

[6] Shoffstall A.J., Ecker M., Danda V., Joshi-Imre A., Stiller A., Yu M., Paiz J.E., Mancuso E., Bedell H.W., Voit W.E. (2018). Characterization of the Neuroinflammatory Response to Thiol-ene Shape Memory Polymer Coated Intracortical Microelectrodes. Micromachines.

[7] Rihani R.T., Kim H., Black B.J., Atmaramani R., Saed M.O., Pancrazio J.J., Ware T.H. (2018). Liquid Crystal Elastomer-Based Microelectrode Array for In Vitro Neuronal Recordings. Micromachines.

[8] Kil D., Carmona M.B., Ceyssens F., Deprez M., Brancato L., Nuttin B., Balschun B., Puers R. (2019). Dextran as a Resorbable Coating Material for Flexible Neural Probes. Micromachines.

[9] Bernardin E.K., Frewin C.L., Everly R., Ul Hassan J., Saddow S.E. (2018). Demonstration of a Robust All-Silicon-Carbide Intracortical Neural Interface. Micromachines.

[10] Sridharan A., Chirania S., Towe B.C., Muthuswamy J. (2019). Remote Stimulation of Sciatic Nerve Using Cuff Electrodes and Implanted Diodes. Micromachines.

[11] Straka M.M., Schafer B., Vasudevan S., Weller C., Rieth L. (2018). Characterizing Longitudinal Changes in the Impedance Spectra of In-Vivo Peripheral Nerve Electrodes. Micromachines.

[12] Vajari D.A., Vomero M., Erhardt J.B., Sadr A., Ordonez J.S., Coenen V.A., Stieglitz T. (2018). Integrity Assessment of a Hybrid DBS Probe that Enables Neurotransmitter Detection Simultaneously to Electrical Stimulation and Recording. Micromachines.

[13] Nicolai E.N., Michelson N.J., Settell M.L., Hara S.A., Trevathan J.K., Asp A.J., Stocking K.C., Lujan J.L., Kozai T.D.Y., Ludwig K.A. (2018). Design Choices for Next-Generation Neurotechnology Can Impact Motion Artifact in Electrophysiological and Fast-Scan Cyclic Voltammetry Measurements. Micromachines.

[14] Sharma M., Gardner A.T., Strathman H.J., Warren D.J., Silver J., Walker R.M. (2018). Acquisition of Neural Action Potentials Using Rapid Multiplexing Directly at the Electrodes. Micromachines.

[15] Xu J., Guo H., Nguyen A.T., Lim H., Yang Z. (2018). A Bidirectional Neuromodulation Technology for Nerve Recording and Stimulation. Micromachines.

[16] Goncalves S.B., Palha J.M., Fernandes H.C., Souto M.R., Pimenta S., Dong T., Yang Z., Ribeiro J.F., Correia J.H. (2018). LED Optrode with Integrated Temperature Sensing for Optogenetics. Micromachines.

[17] Scholvin J., Zorzos A., Kinney J., Bernstein J., Moore-Kochlacs C., Kopell N., Fonstad C., Boyden E.S. (2018). Scalable, Modular Three-Dimensional Silicon Microelectrode Assembly via Electroless Plating. Micromachines.

[18] Hoch K., Pothof F., Becker F., Paul O., Ruther P. (2018). Development, Modeling, Fabrication, and Characterization of a Magnetic, Micro-Spring-Suspended System for the Safe Electrical Interconnection of Neural Implants. Micromachines.

[19] Winter B.M., Daniels S.R., Salatino J.W., Purcell E.K. (2018). Genetic Modulation at the Neural Microelectrode Interface: Methods and Applications. Micromachines.

[20] Shokoueinejad M., Park D.W., Jung Y.H., Brodnick S.K., Novello J., Dingle A., Swanson K.I., Baek D.H., Suminski A.J., Lake W.B. (2019). Progress in the Field of Micro-Electrocorticography. Micromachines.

[21] Barth B.B., Huang H.I., Hammer G.E., Shen X. (2018). Opportunities and Challenges for Single-Unit Recordings from Enteric Neurons in Awake Animals. Micromachines.

[22] Kozai T.D.Y. (2018). The History and Horizons of Microscale Neural Interfaces. Micromachines.

